# Challenges and Opportunities in Causality Analysis Using Large Language Models

**DOI:** 10.3390/e28010023

**Published:** 2025-12-24

**Authors:** Wlodek W. Zadrozny

**Affiliations:** Computer Science & Data Science, University of North Carolina Charlotte, Charlotte, NC 28223, USA; wzadrozn@charlotte.edu

**Keywords:** causality extraction, large language models, LLM, NLP, causality, GPT, Gemini, causality datasets, CNC corpus, causality analysis

## Abstract

This article examines the challenges and opportunities in extracting causal information from text with Large Language Models (LLMs). It first establishes the importance of causality extraction and then explores different views on causality, including common sense ideas informing different data annotation schemes, Aristotle’s Four Causes, and Pearl’s Ladder of Causation. The paper notes the relevance of this conceptual variety for the task. The text reviews datasets and work related to finding causal expressions, both using traditional machine learning methods and LLMs. Although the known limitations of LLMs—hallucinations and lack of common sense—affect the reliability of causal findings, GPT and Gemini models (GPT-5 and Gemini 2.5 Pro and others) show the ability to conduct causality analysis; moreover, they can even apply different perspectives, such as counterfactual and Aristotelian. They are also capable of explaining and critiquing causal analyses: we report an experiment showing that in addition to largely flawless analyses, the newer models exhibit very high agreement of 88–91% on causal relationships between events—much higher than the typically reported inter-annotator agreement of 30–70%. The article concludes with a discussion of the lessons learned about these challenges and questions how LLMs might help address them in the future. For example, LLMs should help address the sparsity of annotated data. Moreover, LLMs point to a future where causality analysis in texts focuses not on annotations but on understanding, as causality is about semantics and not word spans. The Appendices and shared data show examples of LLM outputs on tasks involving causal reasoning and causal information extraction, demonstrating the models’ current abilities and limits.

## 1. Introduction

This article provides *an* overview of applications of Large Language Models (LLMs) to the “causality extraction” problem in Natural Language Processing (NLP). It aims to be conceptually comprehensive but it may be incomplete because the field is changing. It is also, in its conclusion, a position paper about how the field can change: with the appearance of new, very capable language models, the current focus on the *annotations* of causal segments can (should?) be replaced with *semantic analysis* and explainability, and employ different models and different viewpoints.

Large Language Models (LLMs) have been the dominant research theme in Natural Language Processing (NLP) since 2018, when BERT [1] was made available by Google. BERT gave rise to several related models, e.g., SciBERT, DistillBERT, BioBERT, and others. Later OpenAI introduced GPT-2 and with GPT-3.5, we observed the emergence of “intelligent behavior” and the adoption of the ChatGPT app skyrocketed. Since then, we have seen great improvements in several versions of GPT: GPT-4o, o3 (with reasoning+agents) [2,3], and GPT-5 [4] and many interesting research results using publicly available Large Language Models (LLMs) such as LLAMA, Gemma, Gemini 2.5 Pro, Grok, Falcon, Claude, Mixtral, and others [5].

Due to the generative nature of LLMs, they often “hallucinate” the locations of relevant action, cause, or effect passages (discussed in more detail in a subsequent section). Additionally, their relatively shallow contextual understanding can hinder their performance in generating accurate interpretations, particularly for complex domains like political news, e.g., [6,7]. Thus, on the one hand we are seeing tremendous progress in the quality of LLMs, but on the other hand, we cannot be sure of the quality of produced results and their inexplicable occasional total failures. Therefore, we need to address the question of the reliability of LLMs for the causality analysis of texts, which this article realizes.

Causality extraction traditionally involves two key tasks: identifying phrases and clauses that describe causes, actions, and effects and situating these elements within broader contexts. Examples include improving a patient’s condition or explaining the political implications of an event and its causes. In other cases, the task might involve finding causal relations between several related events, described in a newswire article, or diagnosing why a product is not reliable. For illustration, consider the following examples of recognized causal relations:

**Example** **1.**

*(1)*  *It was recognized that any <C> assessment of glycemia in early pregnancy </C> would also result in <E> detection of milder degrees of hyperglycemia short of overt diabetes </E>.*
*(2)*  *<causal-relation> When a <cause> policyholder or insured person becomes sick or hurt </cause>, the company <trigger> pays </trigger> <outcome> cash benefits fairly and promptly for eligible claims </outcome> </causal-relation>.*
*(3)*  *If he had* [*reduced his sugar intake*${]}_{CO}$*, he would be* [*free from diabetes*${]}_{E}$*.*
*(4)*  *[* *[A]* *Timely referral] is indicated] if [* *[CO]* *chronic or recurrent symptoms severely affect the patient’s productivity or quality of life]].*

The differences in notation are apparent and Section 4, Section 6, and Section 7 will return to this problem. In the meantime, we can guess that in (1) <C> stands for “cause” and <E> for “effect”; and in (4), [CO] refers to a condition potentially leading to an action [A] (4) and (2) has <outcome>, leading to a question of whether it might be the same as <E>. Notable is also the fact that causal relations in (1) and (2) do not include any verbs unlike (4). Also note a counterfactual (3). Upon reflection, the vocabulary used in these examples can have multiple meanings. What **are** causes, effects, conditions, triggers, actions, and outcomes? Are effects and outcomes the same thing? What about conditions, e.g., is any vital sign of the patient part of a condition? In other words, where are the boundaries of these concepts drawn? We will return to this issue later in Section 3 and Section 7.

Another example shows causality between events from the FinCausal corpus [8], which will be discussed later in Section 4. In this notation, events marked as <e1> are causes and <e2> are their effects.

**Example** **2.**
* *

*<e2> It was found that total U.S. healthcare spending would be about USD 3.9 trillion under Medicare for All in 2019, compared with about USD 3.8 trillion under the status quo.</e2>*

*<e1> Part of the reason is that Medicare for All would offer generous benefits with no copays and deductibles, except limited cost-sharing for certain medications.</e1>*

**Overview of the article:** The article presents an overview of the field, and then showcases recent findings up to early Fall 2025. It is a personal view informed by the author’s experience and the findings of his students. (The overview part of the article is based on the author’s tutorial at FLAIRS-38, May 2025 [9].) It complements other surveys, e.g., [10,11,12,13] (with still maintained pointers to the relevant articles https://github.com/zhijing-jin/CausalNLP_Papers (accessed on 10 October 2025)), as well as another repository on causality extraction [14] https://github.com/causaltext/causal-text-papers (accessed on 10 October 2025).

The present article highlights challenges in causality extraction using LLMs and suggests strategies to make it better. The next section briefly motivates the extraction of causality-related information from text. This is followed by pointing to the diversity of views on causality (Section 3), which as we show later is reflected in capabilities of LLMs. Section 4 grounds the article in an overview of datasets for causality extraction and provides a summary of the recent research on this topic. Then Section 5 introduces LLMs and discusses their growing capabilities, the lack of common sense, and the inevitability of hallucinations in generative models. Then, Section 6 and Appendix B show examples of successful causality analysis with several GPT and Gemini models, using different views of causality including Aristotelian and counterfactual. We conclude with a discussion of how LLMs can help improve the datasets for causality extraction and perhaps change our views of what the problems in understanding causality in text are.

## 2. Why Care About Extracting Causality-Related Information?

**Figure 1:**
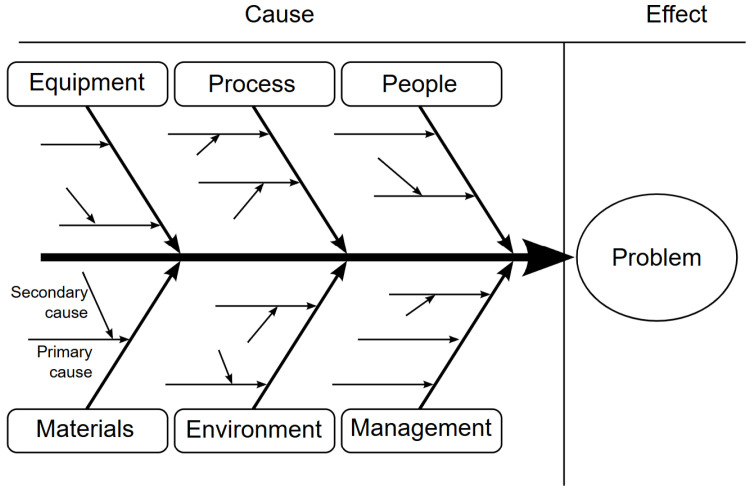
Ishikawa diagrams can be used for cause–effect analysis in complex environments. Source: Wikipedia [16].

We care about finding causal dependencies for several reasons: On the practical side, because human understanding is often based on “mechanical,” action–effect, or cause–effect models. In simpler cases, such as “smoking causes lung cancer,” such relationships are apparent to a human and can also be extracted with high accuracy from simple statements. In more realistic analysis, causal relationships emerge from many factors and typically require a deep knowledge of the domain. For example, to understand and reason about the causes of production defects in industry, or a defeat in a game [15], one can use Ishikawa diagrams [16] to illustrate how the interaction of multiple causes in several categories produces an effect. The overall schema is shown in Figure 1.

**Figure 2:**
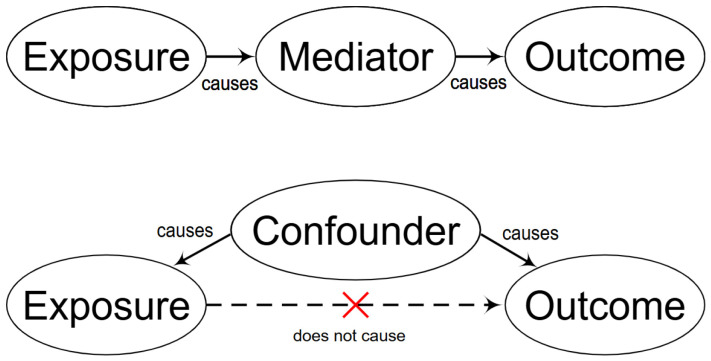
Causal graphs might also help in avoiding errors from data mining by visualizing “mediators” and “confounders.” Source: Wikipedia [16].

There are many—often overlapping—reasons for extracting causality-related information from texts. The general theme seems to be explaining the behavior of complex systems. And an incomplete list would include making informed decisions, e.g., in medicine and business, and predicting outcomes, e.g., in market research and policy making. Such knowledge might also help in avoiding errors from data mining: correlation ⇏ causation; for example, understanding the differences between “mediators” of causality and “confounders”, by building causal graphs $X_{i}⟶X_{j}$, as in Figure 2, where arrows indicate causality and can be grouped into taxonomies as in Figure 1.

Another list of reasons comes from AI itself: Natural Language Understanding (NLU) is not credible without question answering (QA), which itself cannot be conducted in any generality without an account for causality in texts (and other media). Building explainable AI, improving machine learning (including LLMs), and integration with formal reasoning systems could be added to this list. Finally, it is likely a requirement for AGI, that is Artificial General Intelligence [17], which used to be a topic rarely discussed by the general public and became mainstream after the wide adoption of ChatGPT.

## 3. Views on Causality

The topic of causality is vast, e.g., “cause effect relationship” appears about 8 M times in Google Scholar data, and “causality” about 2.8 M times. On the other hand, we obtain “causality extraction” about 900 times, of which 600 after 2021. (This makes this overview somewhat feasible.)

We observed earlier (Section 1) the diversity of annotation schemes in datasets for causality extraction, which presumably reflects the diversity of views of the authors on causality and related terms such as actions, effects, time, etc. (And also reflects the constraints, mainly the cost of annotations.)

To deepen our discussion and better understand the challenges, we need to broaden (slightly) our knowledge of what is or was meant by “causality” by different people and in different contexts. We will do so in the following order:

1. **NLP** adheres to a common sense view of causality as expressed in dictionaries. Tests for causality, administered by text annotators, are used to prepare data for information extraction, question answering, reasoning, and other AI tasks.
2. **Aristotle** introduced the “Four Types of Causes” and argued that all four are *necessary and sufficient* for explanations [18].
3. **Pearl** is mostly concerned with probabilistic causation and is the inventor of the Ladder of Causation (Associational, Interventional, counterfactual) [19,20]. This work is important for reasoning and the quality of explanations.
4. **Other views** will be alluded to mostly through references. This includes “Ontology of Causation” and seven views of causality in medical literature [21] .

### 3.1. Common Sense View of Causality in NLP

This exposition treats “causality extraction” as a separate domain of research in NLP, based on common sense (i.e., dictionary) views of cause and effect in texts. This is more operationally expressed as instructions for annotators, e.g., the “Five Tests for Causality” [22].

1. Why: The example is not causal if the reader is unable to construct a “Why” question regarding the effect.
2. Temporal order: The example is not causal if the cause does not precede the effect in time.
3. Counterfactual: The example is not causal if the effect is equally likely to occur or not occur without the cause.
4. Ontological asymmetry: The example is not causal if the reader can readily swap the cause and effect claims in place.
5. Linguistic: The example is likely to be causal if it can be rephrased into “X causes Y” or “Due to X, Y.”

However, even with these relatively clear instructions, the inter-annotator agreement is between about 30% and 50% [22]. And as we discuss later, in Section 6 and Section 7, one can ask the question whether the tests capture all aspects of interest.

### 3.2. Aristotle’s Four Causes

**Figure 3:**
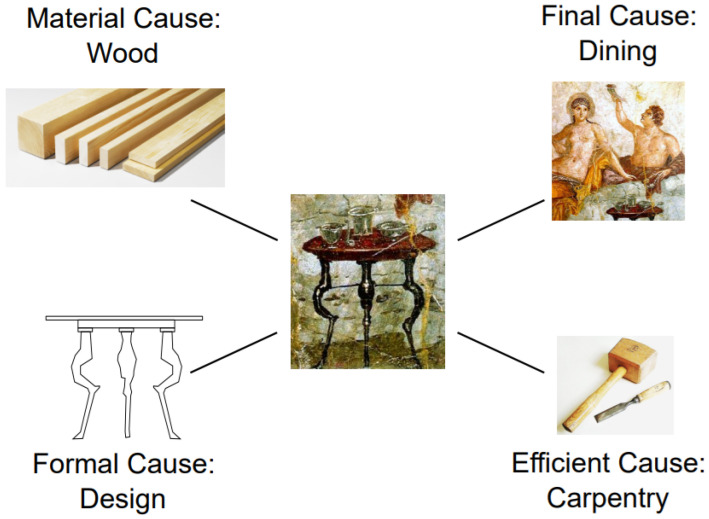
The Four Causes of Aristotle. Source: [23].

According to Aristotle, to fully understand an object or event, one must account for the Four Causes: (1) the material cause, i.e., the material of an object or elements of an event; (2) the formal cause—its form or essence (e.g., the shape or design of the statue); (3) the efficient cause—the agent or process that brings; and (4) the final cause—its purpose. These are exemplified in Table 1 and Figure 3.

We first note that the efficient cause can be abstract (“carpentry”) or concrete (“[t]he agent”). Second, the existence of final causes contradicts Test 2. That is, final causes precede their effects. Such motivating causes can appear in event descriptions, e.g., about a politician starting a smear campaign against the rival “because of the upcoming election.” Thus, future events can influence current events. Of course, we could try to sidestep this problem by talking about a mental representation of “the upcoming election,” but no one in practice would do it when creating annotations. Furthermore, to our knowledge, LLMs have not been trained to reason about mental representations and possible worlds (even though when asked by a series of prompts, they can create, imperfectly, such models). And finally, we see the philosophical (metaphysical) distinction between the form and the matter. We note that philosophy is rarely explicit in NLP, since the focus is almost always on “processing.”

### 3.3. Pearl’s Ladder of Causation

A methodological introduction to Pearl’s view of causation can be found in [19]; this work is expanded to focus on measuring the influence of random variables on possible outcomes within a Bayesian framework [24,25]. Here, its main ideas are presented in Table 2. It is distinguished from the common sense (NLP) and from the Aristotelian views of causality by adding measurements, possibility of experimenting, and reasoning about counterfactuals using a set of previously identified variables.

### 3.4. Other Views of Causation

Given the vastness of the literature on causality/causation, we limit ourselves to the ones with direct, in our view, relevance to the topic “LLMs and causality extraction.” Therefore, we skip discussions of older views on causality (e.g., Hume [26]). We also do not venture into modern methods of understanding causality through data analysis of mediation and interaction of variables, as well as of causality in social networks. For an in-depth treatment of the former, we refer the reader to [27,28]. The last reference also discusses aspects of causality in social networks (spillovers) and has a short discussion of philosophical issues.

However, because of the diversity of annotation schemes for causality in text, we want to mention an article that attempts to organize views of causality in the medical literature into an ontology [21], as represented in the list below (this is our summary of the list from the article). The article also reports that *many studies used the terms “causality,” “causation,” and “cause and effect” as synonyms. However, those who use them may have different meanings and concepts in mind. Many studies appear to define these terms inconsistently or ignore their definition entirely.*

1. **Association**: Causality implied through correlation or co-occurrence, not mechanisms.
  *Example:* Genetic factors are associated with disease susceptibility.
2. **Determinism**: Causation as universal law: if A, then B.
  *Example:* Radiation causes cancer.
3. **Temporal order**: Cause must precede effect in time sequence.
  *Example:* Exposure to a toxin precedes the onset of symptoms.
4. **Disposition**: Objects have inherent powers to manifest effects.
  *Example:* A patient’s disposition to experience adverse effects from a drug.
5. **Causal chain**: Events cause subsequent events in a linear sequence.
  *Example:* A pathogen triggers inflammation, which leads to organ damage.
6. **Influence**: One process modifies the likelihood of another occurring.
  *Example:* Nicotine withdrawal positively influences smoking relapse.
7. **Production**: Cause directly generates or triggers the effect.
  *Example:* A virus produces respiratory symptoms.

### 3.5. Why Care About This Diversity of Views?

As mentioned earlier, causality extraction is not an end in itself; rather, it is a means to improve machine understanding of texts, which again is a means to create better models of reality and, e.g., enable better decision making.

Since LLMs are trained on texts in which all senses of “cause” appear, they will also rely on all these senses when answering questions or performing formal analyses of texts. These views are partly overlapping, but are also distinct. Properties 1–5 do not naturally map into the Aristotle types of causation, nor Pearl’s approach, nor other taxonomies of causal relations, e.g., the seven views of causality in the medical literature [21]. For example, future causes are admissible by Aristotle, but not by NLP annotators, even though we find plenty of news stories explaining how upcoming elections are a cause for dirty political campaigns now. Similarly, prevention—in the case of rare events—contradicts Rule 3 (counterfactual) of Section 3.1. Yet we commonly find texts with examples where skipping an early morning swim prevented being attacked by a shark or taking a child to school prevented being a victim of a terrorist.

With LLMs’ growing importance and their use in both data preparation and causal analysis, we gain, as shown in Section 6, an ability to analyze text documents directly, without the intermediate steps of causality extraction. Moreover, LLMs can answer questions about counterfactuals and Aristotelian causes, even if they are not trained on annotated data for this task. Annotated datasets would still be of value, for example, to check for LLM hallucinations or adjust directions of analysis via better prompting. In addition, numbers (such as probabilities and correlations) are seldom part of annotated data; LLMs could add numerical information to the extracted data and help perform quantitative analysis, including perhaps the interaction of causes as it is performed, e.g., in epidemiology [27] and in other types of research (e.g., [29]).

## 4. Finding Causal Expressions: Datasets and Results

In this section, we provide an overview of causality datasets and published results on causality extraction from texts. As suggested by the preceding examples, this extraction process can operate on different levels: words, phrases, and sentences. Later we are going to see how it can be applied to documents. Furthermore, to be practical, it is necessary to situate the causal elements within broad contexts such as improving patients’ conditions, explaining the political implications of events, or answering questions about a document.

### 4.1. Finding Causal Expressions Within Sentences

Finding “causal” expressions can be viewed as a subspecialty of “relation extraction” in NLP, as shown in sentences in Example 3, taken from the SemEval 2010 Task 8 dataset [30]. Multiple types of relations between entities are of interest and cause–effect is one of them. A recent example survey of “relation extraction” can be found in [31].

**Example** **3.**
* *

*7800 “He was a <e1>trouble</e1><e2>maker</e2> then, a leader in SDS and a trouble maker he remains.”—* *Cause-Effect(e1,e2)*

* *

*7795 “Interspersed in the design are <e1>patterns</e1> derived from a variety of <e2>textiles</e2>.”—* *Entity-Origin(e1,e2)*

However, this article focuses not on all relations, but narrowly on causality. Typically, the first step of this process is finding causal sentences (e.g., [32]). Then, within these sentences, as in all examples above (and below), different methods including LLMs can be used to mark causality expressions by identifying phrases and clauses describing causes, actions, and effects. Less frequently other characteristics, such as percentages or probabilities, are also extracted. This is followed by evaluations on one or more datasets.

We also need to mention causality within the context of multimodal analysis and reasoning, an emerging important sub-field of NLP, with applications to question answering [33] (but this is not a theme of this article).

### 4.2. Charlotte NLP Lab Work on Causality Extraction

The present article is informed to a considerable extent by a decade of research by the Charlotte NLP Lab, that is, my students and collaborators. Our research is summarized in this section and links are provided to the articles, programs, and datasets. There are two streams of this work: (1) causality extraction from medical guidelines; and (2) causality extraction from business texts. They are joined in the reported experiments on transfer learning. Later, Section 5 describes several experiments with LLMs performed in 2024 that will be relevant in the discussion of capabilities of the more recent language models. The next subsection (Section 4.3) attends to other datasets and summarizes other work on causality extraction.

**(1) Causality extraction from medical guidelines:** Clinical Practice Guidelines (CPGs) can contain complex concepts expressed in a complex language. Our work started by extending the results of [34], both by creating new public datasets covering several hundred examples of sentences from hypertension, rhinosinusitis, and asthma guidelines (https://github.com/hematialam (accessed on 15 May 2025)) and annotated primarily for medical conditions, actions, and consequences. Several models were used for causality extraction and example scores include an F1 of 60–70% and accuracy of 85–90+% for Logistic Regression, and for BioBERT [35], the F1 is about 85% and the accuracy is 90–95%. The details of dozens of experiments are given in [36,37,38].

The second dataset in this space is an extract from multiple Gestational Diabetes Guidelines containing about 290 items, which are available at https://github.com/gseetha04 (accessed on 15 May 2025). Here, example results also show the high performance of BERT (various versions) (over 90% accuracy on identifying conditional sentences), which was (as of Fall 2024) better than Llama 3.5 7B and GPT 3.5 and 4; details are provided in [39,40].

**(2) Causality extraction from business texts:** The ORG Dataset (Organizational Behavior) was developed with business school collaborators and has over 14K examples for fine-tuning and over 2.2K for training and testing annotated datasets(in the BIO style) and is available at https://github.com/GoPeaks-AI/text2causalgraph (accessed on 15 May 2025). The dataset comes with a taxonomy, which helps with causality extraction [29,41]. The extraction results are comparable with the ones for the medical guidelines. Namely, 85–91% F1 dataset with BERT (various versions), which were better (as of Fall 2024) than Llama 3.5 7B and GPT-3.5.

Additionally, the results on transfer learning show that when trying to predict performance based on pre-training on different datasets, it turns out that only K-L divergence, measuring the difference in tokens’ distributions, is of value, while—surprisingly—the Kolmogorov–Smirnov test and Wasserstein distance are not predictive. For details, see [39,40,42].

### 4.3. Other Datasets and Studies on Causality Extraction

**Figure 4:**
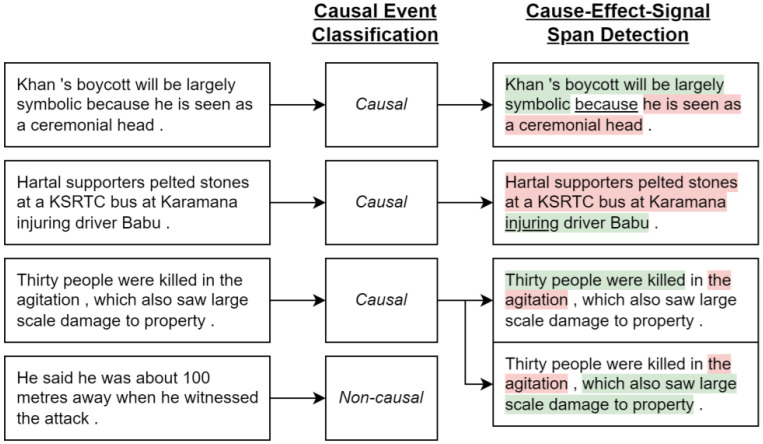
For figure source and an introduction to the **event causality extraction** problem and the CNC dataset, see [45].

**Figure 5:**
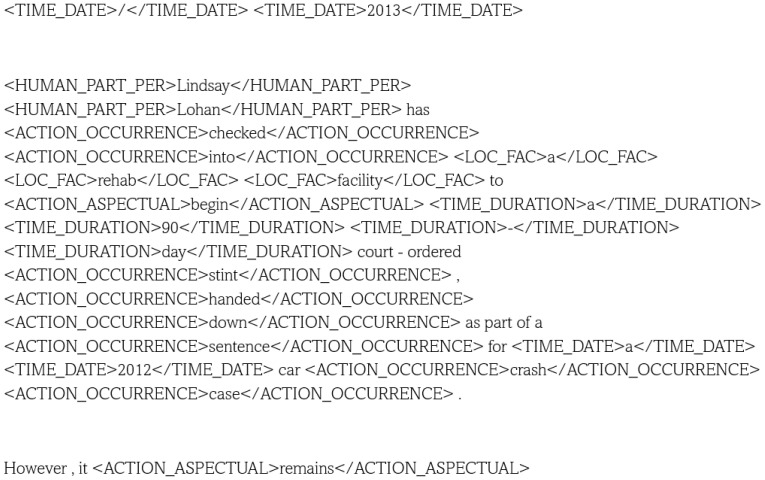
An example of Event Story Line annotations [44] converted by GPT from complex XML to inline annotations for readability’s sake (showing only a small segment of the document).

The experiments reported in this article use a sample of CNC, Chinese News Causality Dataset. This is perhaps the largest causality dataset https://github.com/twinkle121/CNC (accessed on 15 May 2025) and contains 25,629 event mentions and 5569 causal event pairs. Example annotations are shown in Figure 4, and an example result is [43] with an F1 score of 58–82% on the Event Story Line and CNC event causality corpus (however, only 44% on unseen predicates); their architecture incorporates BERT [1]. The mentioned Event Story Line dataset [44] contains 258 documents with temporal relations and causality identification https://github.com/tommasoc80/EventStoryLine (accessed on 15 May 2025). An example annotation in the ESL dataset is shown in Figure 5.

**Figure 6:**
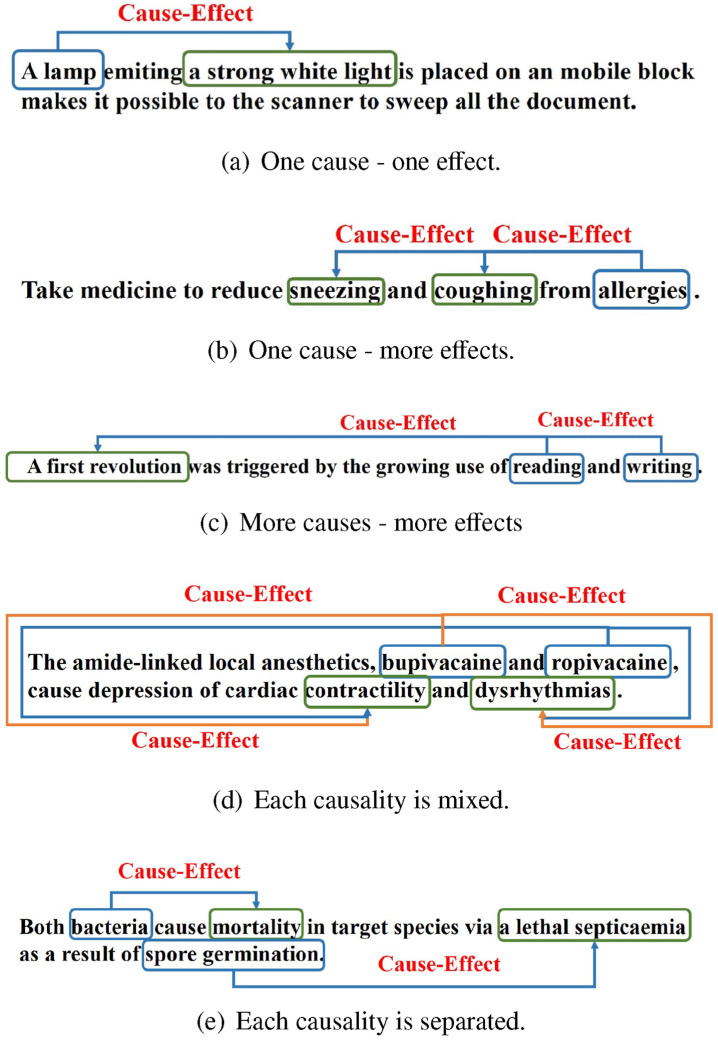
[Wu+24] exemplifies different types of causal relation on the SemEval dataset.

CNC can be compared to the SemEval 2010 Task 8 dataset (mentioned earlier) with 5236 sentences containing 64 embedded causality triplets (see https://huggingface.co/datasets/SemEvalWorkshop/sem_eval_2010_task_8 (accessed on 15 May 2025)). Note that, as shown in Example 3, only entities are being annotated. A recent work involving this dataset is [46], which explores multiple types of neural network architectures, and where yet again, BERT helps produce an F1 score of about 85% . The article also exemplifies multiple types of causality relations, as shown in Figure 6 and earlier in Figure 4.

Finally, we want to mention ExpliCa [47] (https://github.com/Unipisa/explica (accessed on 20 October 2025)), which can be used to evaluate LLMs in commonsense causal reasoning through Pairwise Causal Discovery tasks, i.e., potential causes and effects in pairs of sentences, e.g., as follows:

*“The girl skipped school., The girl had not done her homework.”*

A recent article [47] reports GPT-4o performance of 50–80% depending on a task.

### 4.4. Overall Impression

The easiest thing to notice in the annotated datasets is how different the assignments of causes and effects are. These multiple annotation schemes are not necessarily compatible. Second, the performance of LLMs is stuck at about 85% with various BERT versions often outperforming much larger LLMs (as of April 2025). Third, the annotated datasets are small in comparison with image datasets, which may have one or two orders of magnitude more of examples than the total for all textual causality datasets.

## 5. LLMs, Hallucinations, and Causality

**Figure 7:**
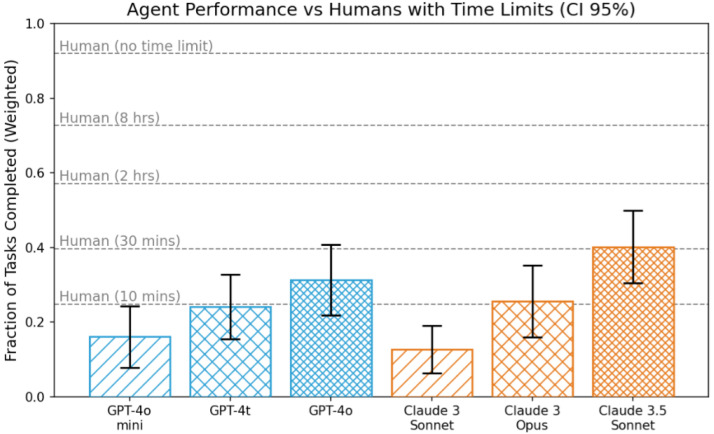
LLMs already help humans on time-consuming tasks. Their performance is changing on a weekly basis (image source: OpenAI [49]). Updates at https://llm-stats.com/.

LLMs are subject to many popular controversies. These include discussions on how intelligent they really are, what will be their impact on labor markets, or whether an AGI will appear in 2027 due to super-exponential growth in capabilities of AI [48]. The underlying question in these discussions is to what extent can we extrapolate from the progress of the last few years, e.g., measured by the performance of various models on several tasks, as illustrated by Figure 7.

There is also quite a bit of skepticism, e.g., performance growth seems to have flattened in 2025 as compared to 2024 https://llm-stats.com/, as measured by the GPQA: A Graduate-Level Google-Proof Q&A Benchmark” [50]. And notwithstanding the progress, the models lack common sense [51].

### 5.1. LLMs Limitations: Hallucinations Occur and Are Unavoidable

The term “hallucinations” in the context of LLMs seems to have entered the vernacular—it is estimated to appear (as of May 2025) at least three million times in the Google search results. However, the label “hallucination” is being applied to many LLM phenomena: disfluency, non-factuality, false confidence, contradicting provided or retrieved (RAG) sources, ignoring prompts, and others; ref. [52] presents a survey and a catalog of problems to which we apply this label. Many empirical methods try to mitigate or investigate hallucinations (surveyed in [53]). Often “fine-tuning” methods are used to reduce hallucinations (improve accuracy). However, such methods can be expensive (e.g., reinforcement fine-tuning [54]).

Importantly, it turns out that current generative LLMs “must hallucinate.” There are several mathematical proofs that LLMs “must hallucinate” [55,56,57,58,59,60]. Intuitively, the space of “suggested facts” (from co-occurrences of phrases) is much larger than the space of established facts. And even though hallucinations can be made statistically negligible [58], the bad news is that larger and more accurate models hallucinate more [3]. Paradoxically, “models can hallucinate with high certainty even when they have the correct knowledge” [59]. And this, of course, can be a serious problem in applications.

### 5.2. Hallucinations and Causality Extraction

For causality extraction, a comparison of fine-tuning and prompting for BERT(s), Llama2, and GPT-4 can be found in [42], e.g., GPT-4 obtains an F1 score of 0.63 with fine-tuning and 0.60 with prompting on the gestational diabetes dataset mentioned earlier. A more recent article [61] discusses causal question answering (QA) and hallucinations. It shows a relatively high accuracy of GPT-4o in English and Spanish on the FinCausal dataset [8], introduced earlier in Example 2. It also offers a few insights: (1) *Prompt optimization and few-shot learning offer some improvements.* (2) *They were insufficient for consistently outperforming extractive methods in FinCausal, suffering from hallucinations.* (3) *In contrast, **fine-tuning generative models** was shown to be essential for minimizing hallucinations and achieving superior performance.* (4) “Both extractive models and generative models struggled at times to extract the correct answer in implicit causal relationships, where explicit causal markers (e.g., “because,” “due to”) were absent.” Perhaps fine-tuning helps LLMs better “understand” what we are looking for by analyzing examples.

## 6. LLMs for Causality Analysis

Given the hallucination problems with LLMs and the accuracy limitations in causality *extraction*, we end on a positive note, showing the promise of current LLMs in causality *analysis*. The presented experiments show that LLMs are capable of sophisticated reasoning about causality.

### Probing LLMs’ Understanding of Causality

**Initial experiments:** Several small-scale experiments were performed by this author in Spring of 2025. A text from the CNC corpus was used (3463_ecbplus.xml.xml—full text is given in Appendix A). It concerned a landslide that struck a quarry, burying a 2000-square-meter plant. Seventeen workers were on site: fourteen were safely evacuated and three remained missing. Over 300 rescuers and multiple teams from various departments were mobilized for large-scale search and rescue operations. Possibly, the landslide was triggered by prolonged dry weather that loosened the mountain slope.

**Figure 8:**
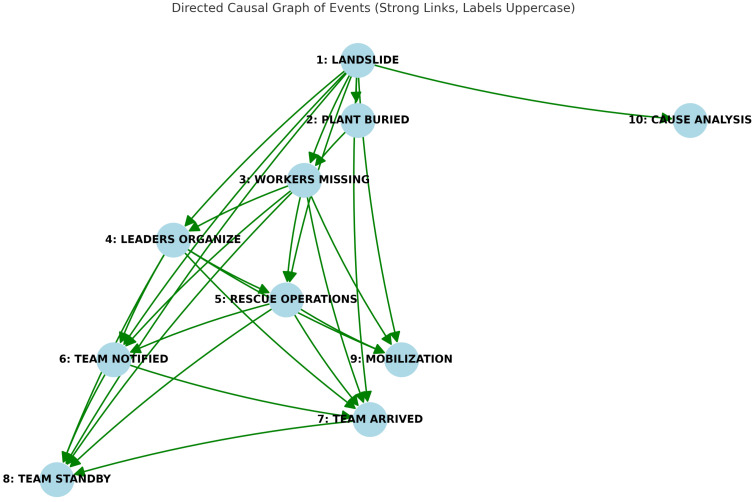
Causal graph for **3463_ecbplus.xml.xml** from the CNC corpus created by GPT-4o in early May 2025. Note that the causes are shown as words or simple phrases in contrast to the causal graph generated by Gemini shown in Figure 9.

**Figure 9:**
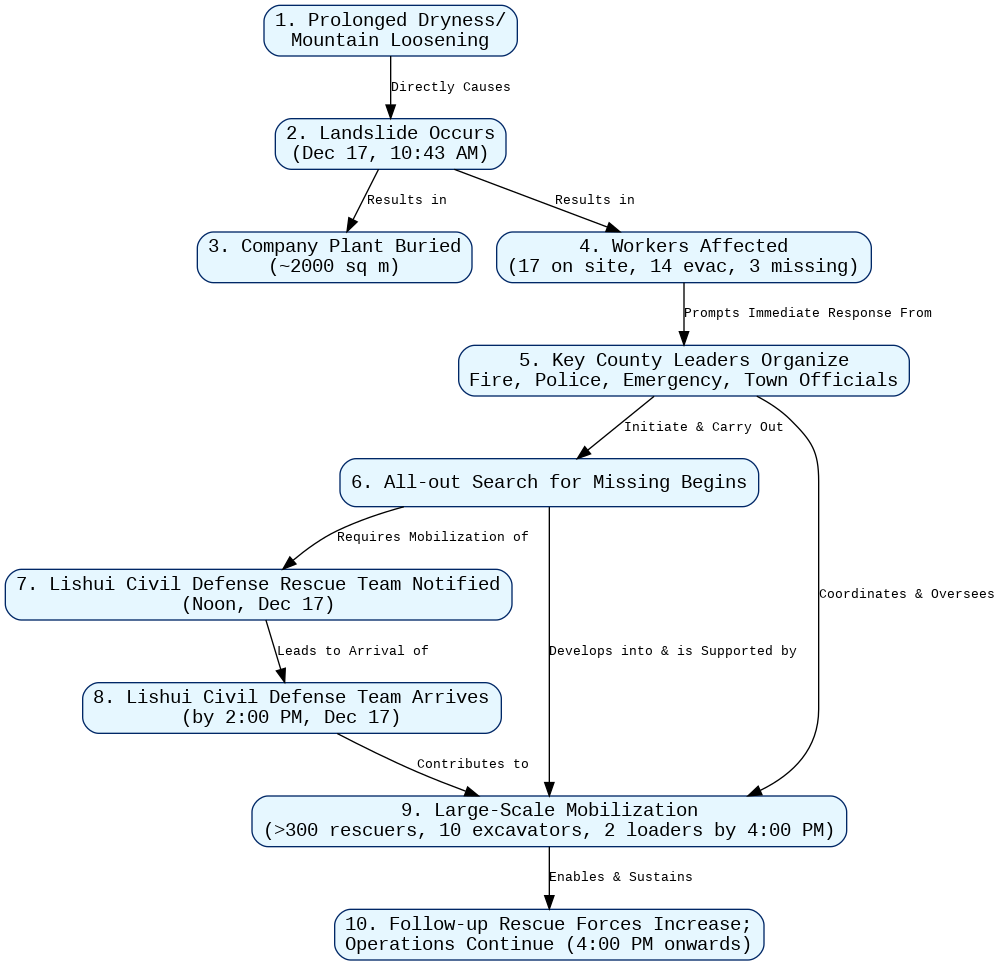
Graph of CNC corpus example **3463_ecbplus.xml.xml** using Gemini Advanced 2.5 Pro; early May 2025. Note that connections between states and events have their causes and relationships described, in contrast to Figure 8 generated by GPT.

The experiment, with GPT-4o and Gemini Advanced 2.5 Pro, showed the potential of LLMs to “understand” causality. For example, both were able to create causal graphs and reason about indirect and direct causes, as well as perform a counterfactual and Aristotelian analysis. The respective causal graphs are shown in Figure 8 and Figure 9. Note that the graphs are relatively correct (except for Node 10 in Figure 8) and informative. The details are given in Appendix A.

**Substantive experiments:** In the Fall of 2025, a larger experiment on a random sample of 25 texts from the earlier-mentioned CNC corpus was performed by this author. The texts were translated into English (by DeepSeek). GPT-5 and Gemini 2.5 Pro were prompted to extract pairs of events and establish causal relations between them. The experiments were conducted using Google Colab Pro and the respective APIs, with default temperature settings. The prompts asked for extracting ten events from each text, ordering them chronologically, and finding direct or indirect causal relations between them. After finding causal relationships, the models were asked to perform a critique of the reasoning of the other LLM.

Some of the produced reports were slightly transformed using a few regular expressions (manually, using Notepad++) because the outputs were not always given in the requested format. Only formatting was affected and not any of the texts, detected causal pairs, or critiques. Such post-processing is not surprising, as online LLMs operate in parallel, possibly direct our requests to different versions of the generative models, and do not always provide consistent outputs. This phenomenon is more problematic for extracting causal relationships via annotation, but seems to have no effect on the semantic analysis in this experiment.

The results are interesting and show very high agreement between the LLMs in their causal analysis. (As contrasted with the relatively low inter-annotator agreement on the annotation task mentioned earlier in Section 3.1.) Gemini 2.5 Pro claimed 19 pairs to be incorrect out of 213 pairs analyzed by GPT-5, i.e., disagreed in about 9% of the cases. GPT-5, in analyzing Gemini 2.5 Pro, viewed 28 of 225 pairs as incorrect, i.e., disagreed in about 12% of the cases. Overall, the quality of the analysis was very high. In manual analysis, no hallucination events or clearly missing causal pairs were detected.

The number of errors is smaller than the number of disagreements. The majority reflected the underlying differences in reasoning. Thus, of the 28 suspect pairs detected by GPT-5, the manual analysis confirmed 13 errors; the other cases were borderline (10 relations) or likely correct (5 relations). For example, in a discussion about a roof collapse accident, GPT critiques Gemini:

“(e4, e5): Incorrect. The response was initiated because of the incident, not because of the confirmation of 14 trapped.”

However, one could argue that the complex response described in the text was due to people being trapped, because a simple roof collapse, with no victims, would not generate such a response.

Notice that not only do we obtain high-quality causal analyses of events and their critiques, but also the arguments/reasoning supporting the detected causes and effects. In other words, the models give us explainability, which would be lacking if the programs were restricting themselves to creating annotations. In the created analyses, one can see a high degree of “common sense knowledge” (at least for the domain of news reports, which seems to follow common templates).

## 7. Concluding Discussion: Overcoming Challenges in Causal Analysis

In this section, we summarize our preceding observations as a list of challenges for causality analysis and causality extraction. Then, using what we have learned about LLMs in Section 6 (and earlier), we propose a few ways of addressing them (which we plan to do in followup work). Of course, this discussion must be hedged by the limitations of the experiments: one domain, one language, two dozen examples, and only about 450 causal pairs. Nevertheless, the results are strongly suggestive of the emerging capabilities of LLMs in understanding causal relations expressed in text.

### 7.1. Challenges in Causality Extraction—What Have We Learned?

The first challenge in causality analysis and causality extraction lies in *conceptualization*, that is, there are many views of causality and the examples and references provided in this article (mainly in Section 3) are by no means exhaustive. The question arises whether different domains (e.g., news events vs. medical) require different ontologies for expressing causation, which seems to follow from some cognitive science experiments [62]. Or, even more, whether they should be different for different sets of experimental data, as seems to be the case in medicine [21].

This is further illustrated by the many datasets for causality extraction and their incompatible representations of causality. These datasets follow different notational conventions and, more importantly, have different (implicit) ontologies.

Furthermore, the causality datasets are small because the manual annotations are expensive to create and high inter-annotator agreements are difficult to achieve. And this is yet another, important, challenge. A third—and related—challenge is the low inter-annotator agreement, and our lack of the ability to question the decisions made by the annotators once the datasets are published.

A fourth challenge has to do with LLMs having difficulty in keeping track of the sequences of annotations, and “ hallucinating” beginnings and ends of annotations. In all interactions with LLMs, one should be aware of hallucinations and keep in mind cautionary examples (e.g., [63]) pointing to the fact that LLMs operate on “well-worn mental shortcuts” and can produce incorrect results, even if challenged. One might hope that symbolic co-processing would eventually help, but at this point, there are no guarantees of correctness.

### 7.2. Can Causality Analysis with LLMs Help Address These Four Challenges?

Based on the example results discussed in the previous section, Appendix B, and in the experiment data made available online, the answer is a tentative “Yes”. Yet in the absence of large-scale analyses, this conclusion is still tentative, although informed by the author’s experience with LLMs and causality extraction [29,38,40,42,64,65].

To start with, different conceptualizations can be used with LLMs, for example, their common sense ones are different, and they approach Aristotelian point of view differently. Still, experiments to confirm or disprove this possibility are yet to be performed.

Can the ontologies which are implicit in annotation instructions, or explicit ones, be used to guide LLMs towards a particular type of analysis? This article is unable to provide an answer. Thus, the first challenge remains open.

Next, let us discuss the challenge of data paucity. In the best case, when the data is unambiguous and follows a pattern, one can fine-tune LLMs to good performance. The creators of datasets often attempt to follow patterns and avoid ambiguity. This suggests that LLM-assisted translation between different dataset formats should be possible. For example, in Figure 8 and Figure 9, we see that causal dependencies are expressed both as short phrases and as events connected by specific actions (based on the common text).

Furthermore, given the high-quality machine translation, it is possible to translate datasets between languages, e.g., from Chinese to English, as we have conducted in our analyzed example. These two steps would help increase the amounts of available data and perhaps enable additional experiments in other languages, e.g., for which no causality data exists.

The paucity of data can also be addressed by using LLMs as annotators (this is not a novel idea). If performed at scale, we could create large datasets by keeping only the items on which they agree, and perhaps analyzing the disagreements, either manually or automatically, for conceptual disagreements. At the minimum, these steps would give us (somewhat?) interoperable datasets and address the third problem: low inter-annotator agreement. Notice that even if the quality of causal analysis by LLMs is better than now, we will still need datasets to measure what LLMs do right and where they fail. As suggested by our examples, with proper setup (good data preparation, precise prompts, etc.), they can reason about causes and effects and find regions of agreement.

In the context of multiple annotation schemes and multiple views of causality, likely producing lower inter-annotator agreements, an obvious question arises as to whether forcing one or more ontologies on LLMs would improve the quality of the new data, i.e., do we need standards for causality analysis that differ for different domains (politics vs. medicine)? Perhaps they should also differ according to applications or goal, e.g., in medicine, often there is no agreement on causes, actions, and effects, resulting in different guidelines [37,42,66,67].

Such standards can be made explicit. For example, causal analysis with LLMs has to be different when used to create treatment guidelines vs. as a tool in patient–physician decision making. The former depends on the perspective of the medical organization creating the recommendations, while the latter happens in the context of existing recommendations, the patient’s situation, and the physician’s knowledge and diagnostic intuitions. For this, LLMs are improving in understanding contexts of requests or analyses. In addition, in these and many other cases, numbers are important, e.g., for Pearl-style analysis mentioned in Section 3.

To summarize, we have presented an overview of challenges of causality extraction from text (lack of agreement on the concept of causation and its representation and the paucity and quality of available data) and suggested how, controlling for hallucinations, Large Language Models can help address the more general problem of causal analysis of text documents and help address these challenges.

Regarding the fourth challenge (i.e., producing correct annotations), one can expect further improvements in the abilities of LLMs. However, one can also ask whether in most practical cases, we can skip causality extraction and simply proceed with causality analysis, as shown in this article.

## Figures and Tables

**Table 1:** The Four Causes with examples [18].

Cause Type	Description
Material	That from which something is generated and out of which it is made, e.g., the bronze of a statue.
Formal	The structure which the matter realizes and in terms of which it comes to be something determinate, e.g., the shape of the president, in virtue of which this quantity of bronze is said to be a statue of a president.
Efficient	The agent responsible for a quantity of matters coming to be informed, e.g., the sculptor who shaped the quantity of bronze into its current shape, the shape of the president.
Final	The purpose or goal of the compound of form and matter, e.g., the statue was created for the purpose of honoring the president.

**Table 2:** Ladder of Causation: Pearl’s three-level Causal Hierarchy (PCH) with representative questions, examples, and ML paradigms. Reproduced from [20].

	Layer (Symbolic)	Typical Activity	Typical Question	Example	Machine Learning
$L_{1}$	Associational P(y∣x)	Seeing	What is? How would seeing X change my belief in Y?	What does a symptom tell us about the disease?	Supervised and Unsupervised Learning
$L_{2}$	Interventional P(y∣do(x),c)	Doing	What if?What if I do X?	What if I take aspirin, will my headache be cured?	Reinforcement Learning
$L_{3}$	Counterfactual $P(y_{x}|x^{\prime },y^{\prime })$	Imagining	Why?What if I had acted differently?	Was it the aspirin that stopped my headache?	

## Data Availability

Pointers to available datasets are given in the text of the article. The code and data used in the experiments are available at https://drive.google.com/drive/folders/1VujYfpQ-6iDuGJut81dpHXnlpqGuHYkP (accessed on 10 December 2025).

## Abbreviations

    The following abbreviations are used in this manuscript:

LLM(s)	Large Language Model(s)
NLP	Natural Language Processing

## Appendix A. Working with GPT-4o on a Longer Text

We use a random example from the CNC corpus mentioned earlier (**3463_ecbplus.xml.xml**). The example is in Chinese; therefore, it needs to be translated into English, which is followed by a causality analysis of English text in the steps below, of which we show only results for steps 1, 4, and 6. The whole conversation is available at https://chatgpt.com/share/685d81d7-0260-800a-a2a2-9ac21226761a (accessed on 10 October 2025).

1. Translation of the text from Chinese to English (here using GPT, earlier in the article DeepSeek was used, to separate translation from interpretation).
2. Semantic translation including annotations.
3. Finding causal verbs.
4. Listing the numbered points of what happened, in temporal order.
5. Causal analysis for each pair of the points.
6. Creation of the causal graph (several attempts).

**GPT Translation:** *On 17 December in Lishui, reporter Zhou Yulong learned from the Publicity Department of Songyang, Zhejiang, that as of 4:00 p.m. on the 17th, more than 300 rescuers, 10 excavators and 2 loaders had been mobilized to the scene to carry out full-scale search and rescue operations. Follow-up rescue forces were still being increased. Pan Saiqiu and other members of the Lishui Civil Defense Rescue Team received the notice at noon and arrived at the site by 2:00 p.m. “Some of our team members have already entered the site,” said Pan Saiqiu. “Because there’s too much rescue equipment, those of us who haven’t entered the site are standing by at any time.” Currently, more than ten rescue teams—including public security, firefighting, emergency response, militia, medical, electrical, telecommunications and civil rescue forces—are carrying out rescue work at the scene. Around 10:43 a.m. on 17 December, a landslide occurred at the quarry of Anluyuan Mechanized Sand Co., Ltd. in Xinxing Town, Songyang County, Zhejiang Province. Initial checks identified 17 workers on site; three were missing, while the others had been safely evacuated. The company’s plant, covering about 2000 square meters, was buried. After the incident, key leaders of the county’s party committee and government immediately organized firefighting, public security, emergency management and Xinxing Town officials to rush to the scene and carry out an all-out search for the missing persons. Rescue operations are still underway. Preliminary analysis suggests the landslide was caused by prolonged abnormal dryness of the weather, leading to loosening of the mountain.*

* *

**Prompt:** take this paragraph and tell me in 10 numbered points what happened, in temporal order.

**Response:**

1. On the morning of 17 December at around 10:43 a.m.  a landslide occurred at the quarry of Anluyuan Mechanized Sand Co., Ltd. in Xinxing Town, Songyang County, Zhejiang Province.
2. The landslide buried the company’s plant, covering approximately 2000 m^2^.
3. An initial investigation identified 17 workers present at the scene; three workers were missing, while the remaining workers were safely evacuated.
4. Immediately after the incident, county party-committee and government leaders organized firefighting, public-security, emergency-management and township officials to rush to the scene and search for the missing persons.
5. Rescue operations were launched with combined forces from public security, firefighting, emergency response, militia, medical, electrical, telecommunications and civil-rescue teams.
6. By noon on 17 December, Pan Saiqiu and other members of the Lishui Civil Defense Rescue Team had been notified of the incident.
7. By 2:00 p.m., Pan Saiqiu’s team arrived at the scene to assist in the rescue efforts.
8. Some team members entered the site, but owing to the large amount of equipment already present, others remained on standby outside.
9. By 4:00 p.m. on 17 December, more than 300 rescuers, ten excavators and two loaders had been mobilized to continue full-scale search and rescue operations.
10. A preliminary analysis concluded that the landslide was likely caused by prolonged abnormal dryness of the weather, which loosened the mountain slope.

### Causality Analysis with Gemini Advanced 2.5 Pro; Early May 2025

We analyzed the same English version of the CNC text using Gemini Advanced 2.5 Pro in early May 2025 [68] https://ai.google.dev/gemini-api/docs/quickstart?lang=python (accessed on 10 October 2025). We present the prompts and the informative parts of the responses, slightly reformatted, with deletions marked by (…). The transcript of whole session is available at https://g.co/gemini/share/cd52a6147a54 (accessed on 10 October 2025).

* *

**Prompt:** analyze this text: *On 17 December in Lishui, reporter Zhou Yulong learned from the Publicity Department of Songyang, (…).*

**Response:**

This text describes an emergency incident and the subsequent rescue efforts. Here’s an analysis:

- **Key Event:** A landslide occurred at the quarry of Anluyuan Mechanized Sand Co., Ltd. **Location:** Xinxing Town, Songyang County, Lishui, Zhejiang Province, China.
- **Date and Time of Incident:** 17 December, around 10:43 a.m. (The year is not specified in the text, but the reporting is from 17 December of that unspecified year).
- **Impact:*** Human Casualties:* 17 workers were on site. 3 are missing. The other 14 were safely evacuated. *Property Damage:* The company’s plant, covering approximately 2000 square meters, was buried.
- **Response Efforts (as of 4:00 p.m. on 17 December):*** Mobilization:* Over 300 rescuers. 10 excavators. 2 loaders. Follow-up rescue forces were still being increased. *Participating Agencies/Groups:* A multi-agency response is underway, including: (…)
- **Quoted Individual:*** Pan Saiqiu (Lishui Civil Defense Rescue Team):* Confirms his team’s arrival by 2:00 p.m. and mentions that some members have entered the site, while others are on standby due to the amount of rescue equipment.
- **Source of Information for the Report:** Reporter Zhou Yulong learned the details from the Publicity Department of Songyang, Zhejiang.
- **Preliminary Cause of Landslide:** Prolonged abnormal dryness of the weather, leading to the loosening of the mountain.
- **Overall Tone:** The tone is factual and urgent, focusing on the details of the incident and the scale of the rescue operation. It highlights the immediate and comprehensive response from various authorities and volunteer groups.
- **Missing Information:** The specific year of the incident is not mentioned. The condition of the three missing workers. Specific details about the “prolonged abnormal dryness” (e.g., duration).
- **In summary**, the text provides a snapshot of an active emergency situation—a landslide at a quarry—detailing the immediate aftermath, the significant rescue operation launched, the human impact and the preliminary suspected cause. It emphasizes a coordinated, large-scale response from multiple official and civil organizations.

**Prompt:** Now extract 10 key events and put them in chronological order

**Response:** (We skip it, since Figure 9 adequately represents the extracted events)

* *

**Prompt:** take each pair of bullets and tell me if bullet i caused bullet j and why.

**Response (Segment of analysis)**

      (…)

2. Did Bullet 2 (Landslide) cause Bullet 3 (Plant buried)?

      Yes. Why: The landslide is the physical event that directly resulted in the company’s plant being covered by debris.

      (…)

**Prompt:** --what about indirect causation e.g., 1 to 10 could you investigate these too.

**Prompt: **--now create a directed labeled graph of these causal relations (Note. Three attempts to create the graph did not work properly and no .png file was produced).

* *

**Prompt:** --can you create python code to generate this graph

* *

**Response:** Note: The last prompt resulted in an informative graph shown in Figure 9.

* *

We observe that, with no training examples, both analyses provide a reasonable analysis of causes and effects in the text and both systems can explain their reasoning. No hallucinations are apparent in the event-by-event causality analysis. These analyses are not identical, e.g., the cause of the landslide is provided by Gemini but not by GPT. The graphs in Figure 8 and Figure 9 are different, but so are the representations of causality described earlier: in Example 3 and Figure 6, causal relationships are between words or phrases, while in Figure 4, they are between clauses or events. And given both the lack of a unified perspective on causality and the relatively low inter-annotator agreement mentioned earlier in Section 3.1, using LLMs directly for causality analysis—without the causality extraction—might give us better ways of analyzing and extracting information from text. In particular, as shown in the next subsection, we can ask for analyses with different perspectives. The old and new annotated datasets would still be of value, as a check for LLMs hallucinations, or to guide directions of analysis via better prompting.

## Appendix B. Four Causes and Counterfactuals

In these, very preliminary, experiments both Gemini and GPT were asked to provide both a counterfactual causal analysis and an analysis using the Aristotelian categories. All analyses by GPT and Gemini are given in the shared data (https://drive.google.com/drive/folders/1VujYfpQ-6iDuGJut81dpHXnlpqGuHYkP (accessed on 10 December 2025)) and they are easily followed in the chats at these links (https://g.co/gemini/share/cd52a6147a54 (accessed on 10 December 2025), https://chatgpt.com/share/685d81d7-0260-800a-a2a2-9ac21226761a (accessed on 10 December 2025)). A comparison of Aristotelian and counterfactual analysis will be given in a future work. In this Appendix, we provide a summary illustrating the spirit of the analysis.

Let us start with Aristotle. Since Final Causes are not given in news reports, should we regard the answers below as unjustified speculations (“hallucinations”) or an indication of plausible, common sense reasoning?

GPT speculated that the Final Cause(s) possibly were as follows:

    “Economic aim: extract construction aggregate at lowest cost; Corporate mandate: maximize output, minimize downtime; Local demand for sand/concrete in Zhejiang’s building sector.” And it commented “Teleologically, the quarry wall was shaped the way it was so that large volumes of rock could be removed rapidly and cheaply. Reduced catch-bench spacing or steeper faces keep haul distances short, satisfying the final cause (profit), but at the expense of higher geotechnical risk.”

On the other hand, Gemini made the following warning:

    “The final cause, or *telos*, refers to the end, purpose, or the sake for which an event occurs. This is challenging for natural events which don’t have intention.”

### Appendix B.1. FourCauses Analysis by Gemini 2.5 Pro

**The Event:** A landslide at the quarry of Anluyuan Mechanized Sand Co., Ltd. in Xinxing Town, Songyang County, Zhejiang Province, on 17 December.

1. **Material Cause (What is it made of?)**
  The material cause refers to the underlying matter from which the event or object is constituted. For the landslide:
  - **The Mountain Itself:** This includes the rock, soil, earth and any other geological materials that comprised the slope before it failed.
  - **Water (or lack thereof, then its presence indirectly):** While the *prolonged dryness* is cited as a factor for loosening, the actual movement in many landslides involves the interaction of water with the soil/rock. In this specific case, the dryness led to “loosening,” implying a change in the material properties that made it susceptible to movement.
  - **The Company’s Plant and Equipment:** These became part of the debris and the consequence of the landslide, but the primary material of the *landslide itself* is the earth material.
  Essentially, the material cause is the physical “stuff” that moved and was affected.
2. **Formal Cause (What is its form or essence?)**
  The formal cause is the pattern, model, or structure that defines the event; what makes it recognizable as a “landslide.”
  - **The Definition of a Landslide:** The form is the rapid movement of a mass of rock, debris, or earth down a slope.
  - **The Specific Characteristics of** ***This*** **Landslide:**
    - Occurred at a quarry.
    - Buried a plant of approximately 2000 square meters.
    - Involved a specific volume and type of material (though not detailed in the text beyond “mountain”).
    - Had a specific failure mechanism (e.g., a slump, a debris flow, a rockfall–the text doesn’t specify, but the “loosening” suggests a less cohesive failure).
  - **The Shape and Trajectory of the Moving Mass:** The way the earth detached, moved and came to rest.
  The formal cause is what allows us to identify this event as a “landslide” rather than, say, an earthquake or a flood.
3. **Efficient Cause (What brought it into being?)**
  The efficient (or moving) cause is the agent or process that initiated the event. This is often what we think of as the “cause” in modern scientific terms.
  - **Primary Efficient Cause (as per text):** “Prolonged abnormal dryness of the weather, leading to loosening of the mountain.”
  - **Contributing/Underlying Efficient Causes (Implied or General):**
    - **Gravity:** The constant force pulling the loosened material downwards.
    - **Geological Conditions:** The inherent properties of the rock and soil, slope angle and geological structure of the mountain at the quarry site.
    - **Quarrying Activities (Potentially):** The landslide occurred *at a quarry*. This human activity could be a significant contributing efficient cause.
    - **The specific moment of failure:** Some final, perhaps imperceptible, trigger that caused the already loosened material to give way.
  The efficient cause is the “why it happened now” and “what made it happen.”
4. **Final Cause (Telos: What is its purpose or end?)**
  The final cause, or *telos*, refers to the end, purpose, or the sake for which an event occurs. This is challenging for natural events which don’t have intention.
  - **In a Purely Naturalistic Sense:**
    - **Achieving a More Stable State:** From a physics perspective, the materials moved to reach a lower energy state, a more stable equilibrium under the force of gravity.
    - **The Natural Process of Erosion:** Landslides are a natural geological process that shapes the Earth’s surface over time.
  - **From a Human Perspective (Consequences, not “Purpose’):**
    - The text describes the *consequences*: workers missing, a plant buried, a large-scale rescue operation.
    - It’s important not to anthropomorphize the landslide as having an intention to cause harm.
  The human response (rescue) has its own final causes (saving lives, understanding the event).

### Appendix B.2. Causal Analysis Using Aristotle’s Four Causes—GPT-o3

1. **Material + Formal Causes:** Supplied the latent vulnerability:
  - Weak rock.
  - Over-steep configuration.
2. **Efficient Cause:** Acted on 17 December to actualise that vulnerability:
  - Crack propagation → block detachment.
3. **Final Cause:** Explains why the slope was engineered so aggressively:
  - Economic rationale shaped both formal geometry and tolerance for risk.
  Aristotle’s framework clarifies that addressing only the efficient cause (e.g., blaming drought) is insufficient.
4. **Preventive Interventions by Causal Type:**
  - **Material fixes:** rock bolting, shotcrete, drainage to improve inherent strength.
  - **Formal fixes:** redesign bench angles, add catch benches, flatten overall slope.
  - **Efficient controls:** drought monitoring, blast vibration limits, continuous slope radar.
  - **Final-cause alignment:** integrate safety margin into the quarry’s business objectives so the purpose itself no longer incentivises risky geometry.
5. **Conclusion:** By tackling all four causal dimensions, future failures of this type become far less probable.
